# Pathological Features and Prognostication in Colorectal Cancer

**DOI:** 10.3390/curroncol28060447

**Published:** 2021-12-13

**Authors:** Kabytto Chen, Geoffrey Collins, Henry Wang, James Wei Tatt Toh

**Affiliations:** 1 Discipline of Surgery, Faculty of Medicine and Health, The University of Sydney, Westmead 2145, Australia; geoffrey.collins@health.nsw.gov.au (G.C.); henry.wang@health.nsw.gov.au (H.W.); 2 Division of Colorectal Surgery, Department of Surgery, Westmead Hospital, Westmead 2145, Australia

**Keywords:** pathological features, molecular markers, colorectal cancer, prognosis, survival

## Abstract

The prognostication of colorectal cancer (CRC) has traditionally relied on staging as defined by the Union for International Cancer Control (UICC) and American Joint Committee on Cancer (AJCC) TNM staging classifications. However, clinically, there appears to be differences in survival patterns independent of stage, suggesting a complex interaction of stage, pathological features, and biomarkers playing a role in guiding prognosis, risk stratification, and guiding neoadjuvant and adjuvant therapies. Histological features such as tumour budding, perineural invasion, apical lymph node involvement, lymph node yield, lymph node ratio, and molecular features such as MSI, KRAS, BRAF, and CDX2 may assist in prognostication and optimising adjuvant treatment. This study provides a comprehensive review of the pathological features and biomarkers that are important in the prognostication and treatment of CRC. We review the importance of pathological features and biomarkers that may be important in colorectal cancer based on the current evidence in the literature.

## 1. Introduction

Colorectal cancer (CRC) is a common malignancy and one of the leading causes of cancer death worldwide [1]. It represents a heterogenous group of tumours that display diverse clinicopathological features and outcomes [2]. The prognosis of CRC patients varies greatly between patients with 5-year survival rates ranging from 90% to 10% depending on stage and other factors [3]. 

Prognostication of colorectal cancer (CRC) relies mainly on cancer stage as defined by the Union for International Cancer Control (UICC) and American Joint Committee on Cancer (AJCC) TNM staging classification. However, there are considerable differences in clinical outcomes and prognosis within patients of the same pathological stage, especially within the intermediate stages of CRC (stages II and III) [4,5]. Further risk stratification may be important to identify patients at a high risk of recurrence or metastases and to guide prognosis and management. 

Histological features, such as tumour budding, perineural invasion, apical lymph node positivity, lymph node yield, lymph node ratio, and molecular features such as microsatellite instability (MSI), Kirsten rat sarcoma virus (KRAS), v-RAF murine sarcoma viral oncogene homolog B (BRAF), and caudal type homeobox 2 transcription factor (CDX2) have been used to guide prognostication and optimise adjuvant treatment, but there is no consensus on their role. This review summarises the available evidence in the literature pertaining to pathological features and biomarkers that are important in the prognostication of CRC patients.

## 2. Literature Search

Two databases (MEDLINE and Embase) were searched using the following search strategy: “colorectal cancer” or “colorectal neoplasms”, “tumour stage”, “nodal stage”, “metastasis” or “distant metastasis”, “tumour size”, “BRAF” or “BRAF mutation”, “KRAS” or “KRAS mutation”, “tumour budding”, “tumour location”, “tumour infiltrating lymphocytes”, “CDX2 mutation”, “lymph node yield”, “lymph node ratio”, “apical lymph node status”, “perineural invasion”, “circumferential resection margin”, “tumour grade”, “lymphovascular invasion” and “prognosis”. After excluding non-relevant studies, 1447 abstracts were identified through MEDLINE and Embase and additional studies were found from hand-searching references, with 276 studies included in this review.

## 3. Pathological Features

### 3.1. Overview

In this study, pathological features have been divided into the TNM stage, molecular biomarkers, and histological features. TNM staging classification include tumour stage, nodal stage, and distant metastasis. TNM is the most important pathological classification in all international CRC guidelines [1,2,6,7,8,9,10]. 

Molecular biomarkers, include KRAS, BRAF, MSI, and CDX2. Alongside Consensus Molecular Subtypes (CMS), a gene transcriptome-based classification system defining four disease entities of CRC (CMS 1–4) and capturing CRC heterogeneity at the genetic level [11], studies have shown that the combination of molecular biomarkers with CMS has the potential to predict response to both chemotherapy and immunotherapy in CRC [11,12,13]. Furthermore, KRAS mutations, BRAF V600E mutations, MSI, and the CpG island methylator phenotype (CIMP) status [12,14] are closely correlated with CMS. The above molecular biomarkers have been reviewed in this study but as CMS is a genetic rather than pathological classification, CMS was beyond the scope of this study. 

Histological features include tumour size, tumour budding, tumour infiltrating lymphocytes (TIL), lymph node yield (LNY), lymph node ratio (LNR), apical lymph node (ALN) status, perineural invasion (PNI), circumferential resection margin (CRM), lymphovascular invasion, and tumour grade. 

### 3.2. TNM Stage

Following the diagnosis of CRC, clinical and pathological staging is essential to determine the local and distant extent of disease, which in turn provides a framework for determining prognosis and therapy. The AJCC-UICC tumour node metastasis (TNM) staging system (8th edition, 2017) remains the gold standard for the prognostication of newly diagnosed CRC. Originally developed to predict prognosis in 1968, the TNM staging system has since expanded in scope to guide management, which is reflected in numerous international guidelines [15]. The TNM system classification provides strong prognostication for patients with early (stage I) and late (stage IV) disease. For patients with stage II and III disease however, there is more heterogeneity in their prognosis and outcomes [2]. 

#### 3.2.1. Tumour (T) Staging

Tumour staging in CRC has been shown to independently and negatively influence survival [16,17,18,19,20,21,22,23,24]. In multiple population-based studies, a higher T stage is associated with worse 5-year overall survival (OS) (T3 87.5%, T4 71.5%) [20,22,23,25], declining to 46% in T4b tumours [26]. A higher T stage is also correlated with poorer disease-free survival (DFS) [27,28] and relapse [20,21,22,24]. Tsikitis et al. demonstrated a three-fold increased risk of recurrence in T4 tumours compared with T3 tumours [29]. Higher T stage is associated with increased incidence of nodal metastasis, distant metastasis, and diagnosis in the emergent setting [20,24,30].

#### 3.2.2. Nodal (N) Staging

Regional lymph node involvement is considered the second strongest predictor of outcome in CRC, after distant metastatic spread [20,21,25,31,32]. Regional lymph node involvement is associated with the T stage and histological grade of the primary tumour [33]. Five-year OS in node positive patients ranges from 30–60%, compared to 70–90% in node negative disease [34,35]. Recurrence rates in node positive CRC patients are around 30–35% [36,37], with the majority of recurrences occurring in the first three years following surgical resection [37]. A higher count of involved lymph nodes and reduced lymph node yield (<12) is associated with a worse prognosis [6,38,39,40].

The AJCC-UICC TNM classification stratifies nodal involvement according to the number of involved lymph nodes [6]. While there is emerging evidence on the increasing role of lymph node harvest [39,41], apical lymph node [42,43] and lymph node ratio [44,45], they are not currently included in current nodal staging [6]. Nodal involvement is an indication for adjuvant therapy to reduce the risk of distant metastasis [1,10,46,47]. Adjuvant chemotherapy decreases the absolute risk of death by 10–20% and risk of recurrence by 40% in node positive disease [1,37].

#### 3.2.3. Metastasis (M) Staging

The presence of distant metastasis at diagnosis (stage IV) remains the strongest predictor of prognosis and outcome [6]. A total of 35 to 50% of patients present with distant metastasis at diagnosis, and this confers a 5-year OS of less than 10% [6,48]. Chemotherapy is used mainly with palliative intent, and increases median survival from 5 to 18 months [49,50]. The most common site of distant spread is the liver due to the portal venous drainage of the intestinal tract, followed by lungs, bone, and other sites [51,52,53]. Distal rectal tumours may initially metastasize to the lungs as the inferior rectal veins drain directly into the inferior vena cava rather than the portal venous system [51,52,53].

**Core Tip:** TNM classification is the most commonly used system for prognostication and to guide adjuvant therapy.

### 3.3. Molecular Biomarkers

#### 3.3.1. BRAF

BRAF is a proto-oncogene that encodes the B-RAF protein kinase, a vital component of the mitogen-activated protein kinase (MAPK) pathway [54]. The MAPK pathway in turn plays an essential role in cellular proliferation, differentiation, survival, and apoptosis [55]. BRAF mutation (BRAF-mt) occurs in approximately 11% of all CRC, and plays a key role in tumorigenesis [54,56]. While there are around 30 different BRAF mutations, the V600E mutation is most common, accounting for 90% of all BRAF mutations in CRC [57]. The importance of the BRAF status on prognostication of colorectal cancer remains controversial [14,58,59], though current evidence leans towards poor prognostication [54,56,60,61,62,63,64]. Patients with BRAF-mt CRC tend to be female and older in age at diagnosis. There is an association with poor differentiation, mucinous histology, and proximal location in the colon [55,56,65]. Its utility in prognostication varies according to stage, [56,66,67] and may be affected by the MSI status [60,61,62,63].

The evidence for BRAF mt as a poor prognostic indicator is strongest in metastatic colorectal cancer (mCRC), with worse OS and resistance to EGFR inhibitors [66,68,69,70,71]. A pooled analysis of the CAIRO [72], CAIRO2 [73], COIN [74], and FOCUS [75] studies by Venderbosch et al. in 2014 highlighted worse OS in BRAF-mt mCRC (HR: 1.91; 95% CI 1.66–2.15) and worse progression free survival (PFS) [66]. This is reflected in multiple other studies [65,68,76]. In stage I CRC, there is little evidence that BRAF-mt influences survival [67].

The most significant area of contention lies in stage II and III CRC. Large population studies show mixed evidence but suggest that BRAF is associated with worse prognosis [14,77]. MSI status may influence the utility of BRAF in prognostication. There is conflicting evidence on the relationship between BRAF, MSI, and prognosis [61,63,64]. A 2017 retrospective analysis of the results from the PETACC8 [78] and N0147 [79] trials by Taieb et al. showed BRAF-mt was associated with a significantly shorter time to recurrence (TTR), shorter survival after relapse (SAR), and worse overall survival (OS) in microsatellite stable (MSS) patients (but not MSI patients) with stage III disease [63]. In contrast, a 2016 study by Cuba et al. showed BRAF-mt to be an independent poor prognostic factor in stage II and III MSI disease with regards to CSS but not OS [64]. Further studies suggest BRAF-mt is an independent risk factor for poor prognosis, unaffected by MSI status [80,81,82]. In a 2017 retrospective study, Li et al. examined the effect of BRAF-mt on stage II CRC patients who were not treated with chemotherapy, and showed BRAF-mt to be an independent risk factor for poor prognosis, but interestingly did not find the MSI status to influence prognosis. In this cohort of stage II CRC patients, the authors found that combining the BRAF mutation status with KRAS and PIK3CA mutational status increased the sensitivity in predicting PFS and OS compared to the BRAF mutation alone (ROC AUC 0.65 *p* < 0.002 vs. ROC AUC 0.54 *p* = 0.392) [81]. Thus, the accuracy of prognostication may be increased when BRAF testing is considered in combination with other biomarker tests (KRAS and PIK3CA) [81]. BRAF-mt in high MSI (MSI-H) CRC usually indicates sporadic CRC whereas patients with MSI-H BRAF wild type (BRAF-wt) CRC should be tested for Lynch Syndrome [1,9].

**Core Tip:** BRAF mutation is currently listed as an additional prognostic factor in AJCC-UICC staging guidelines [6]. Current ESMO [1], NICE (UK) [8], and Australian [83] guidelines do not recommend routine BRAF testing in non-metastatic CRC patients, except in MSI-H CRC to distinguish between sporadic and familial (Lynch syndrome) cases. In metastatic CRC, there is stronger evidence for the utility of BRAF testing, and this is reflected in current international guidelines [7,10,46,47]. The ESMO (2014), NICE (2020), and Australian (2017) guidelines recommend testing for BRAF mutation in metastatic colorectal cancer for prognostication and determination of response to anti-EGFR therapies [9,46,47]. BRAF-V600E mutation indicates resistance to anti-EGFR therapy. Patients with mCRC and BRAF-V600e-mt RAS-wt tumours may benefit from the addition of BRAF inhibitors to their adjuvant therapy [7,8,9,46].

#### 3.3.2. KRAS

KRAS is a proto-oncogene that encodes for the K-Ras protein, a vital component of the mitogen-activated protein kinase (MAPK) pathway [84]. KRAS mutation (KRAS-mt) occurs in approximately 40% of all CRC, with a reduced prevalence in the African population (approximately 21%) [85]. KRAS mutations contribute to unregulated cell growth, ultimately leading to the expansion of tumour cell growth [85,86]. KRAS mutations allow cancer cells to grow in lower glucose concentrations than those required for the growth of normal cells [87]. 

While KRAS mutations are a strong predictor of resistance to anti-EGFR therapies, [88] their role in prognostication remains unclear, especially for stage II and III CRC [89]. KRAS-mt CRC patients tend to be female, of mucinous histology and are more likely to be right-sided tumours [90,91]. Some evidence suggests the tendency for poor prognostication may be affected by the MSI status [63,91]. Overall, the evidence in the non-metastatic setting remains inconsistent [85,89,92,93].

In the metastatic setting, with KRAS-mt has been associated with a lower OS and relapse-free survival (RFS) [92,94,95,96]. A pooled analysis of 1239 mCRC patients demonstrated reduced OS (HR 1.41, *p* < 0.001) and PFS (HR 1.2, *p* = 0.03) compared to the KRAS wild type (KRAS-wt) cohort [71]. KRAS-mt mCRC are resistant to anti-EGFR therapy, and this likely contributes to their worse prognosis [88,92].

In the non-metastatic setting, the evidence is less than clear. The QUASAR study found KRAS was associated with an increased risk of recurrence [97]. The 1998 RASCAL population-based study found that only KRAS mutations of pG12V on Codon 12 were significantly associated with poorer OS and DFS [98]. This was verified by the RASCAL II study in 2001 [99]. The N0147 trial in 2014 examining stage III CRC patients undergoing FOLFOX +/− cetuximab showed that KRAS mutations in both codon 12 and 13 were associated with a reduction in DFS [79]. While KRAS-mt CRC are more likely to be right-sided, there have been some studies that have demonstrated that KRAS may be associated with reduced survival in left-sided CRC (not right-sided) [100,101].

There may be an interaction between the KRAS and MSI status. Nash et al. reported significantly higher mortality in patients with MSS KRAS-mt, with a 5-year OS of 55% in KRAS-mt compared with 68% in KRAS-wt. However, this association was significant only in stage I and II disease, and lost its significance in stage III and IV disease [90]. These findings have been supported by the results of the studies by Eklof et al. and Taieb et al., who found reduced CSS in the KRAS-mt MSS cohort [63,102]. However, a study by de Cuba et al. reported the opposite, suggesting that MSI-H KRAS-mt CRC patients were associated with significantly reduced CSS [64].

There have also been studies that have reported that KRAS has no prognostic influence. Roth et al. found no impact on OS (HR = 1.05; 95% CI: 0.85–1.28; *p* = 0.66) and RFS (HR = 4.99; 95% CI: 0.65–3.91; *p* = 0.31), even when patients were stratified by stage or MSI status [91]. These findings were replicated in other studies [84,97,103,104]. Overall, the true prognostic role of KRAS mutation in CRC survival remains uncertain and is not currently used to guide prognosis or adjuvant therapy in the non-metastatic setting.

**Core Tip:** KRAS mutation is currently listed as an additional prognostic factor in AJCC-UICC staging guidelines [6], NICE [47], Australian Cancer Care [9], and ESMO [46] guidelines on colorectal cancer. KRAS testing is important in patients with mCRC as KRAS-mt tumours do not respond to anti-EGFR adjuvant therapy [7,8,9,46]. In non-metastatic CRC, testing for KRAS is not recommended in routine workup of CRC due to a lack of evidence in its utility for prognostication and determination of adjuvant therapy [1,9,47].

#### 3.3.3. MSI

MSI is characterised by frameshift mutations in microsatellite regions [105]. High MSI (MSI-H) occurs in up to 15% of all CRCs, and occurs either due to sporadic mutation (epigenetic inactivation of hMLH1) or in the setting of hereditary nonpolyposis colorectal cancer/Lynch Syndrome [105]. CRC patients without MSI are usually referred to as MSS.

CRC patients with MSI-H tend to be younger, diagnosed at an earlier stage, associated with sessile serrated type, and are more likely to be right-sided [106,107]. Histological features associated with MSI-H include mucin producing tumours, signet-ring cell differentiation, medullary carcinoma, and increased tumour-infiltrating lymphocytes (TILs).

Initially not considered as a strong prognostic factor in 1999 [108], several meta-analyses have shown that MSI is associated with better prognosis [68,109,110,111] and may be important in prognostication in CRC [4], particularly early stage CRC (especially in stage II) [109,110,111]. The meta-analysis by Popat et al. examined 1277 MSI-H CRC patients of all stages, and identified a 35% reduction in risk of overall survival (HR 0.65, 95% CI 0.59–0.71) [110]. An updated meta-analysis by Guastadisegni et al. similarly demonstrated an improved OS, DFS, and DSS in 1972 CRC patients of all stages [111]. The protective effect of TILs in MSI-H CRCs may be protective against dissemination [112]. The prognostic influence of MSI-H in mCRC is less well understood, [60] with several studies demonstrating worse prognosis in the metastatic setting [66,68,113].

Several studies have reported no effect of MSI in prognostication, with some studies showing worse prognosis [114,115,116,117,118,119,120,121]. Preclinical and clinical data have shown that MSI-H CRCs may be resistant to 5-FU therapy, especially in the metastatic setting [109].

**Core Tips:** While the main role of MSI testing is to help identify patients at risk of Lynch syndrome, [10,46] MSI-H CRCs are immunogenic and may be important in prognostication [1,7,9,10,46,47,122]. MSI status may be useful in guiding adjuvant treatment in stage II CRC [1]. Studies have reported resistance to 5-FU therapy particularly in mCRC [9]. Immunotherapy plays an important role in MSI-H mCRC. Programmed cell death receptor 1 (PD-1) and/or cytotoxic T-lymphocyte associated protein 4 (CTLA-4) inhibitors are recommended following the failure of first line cytotoxic chemotherapy [8,9,46]. Furthermore, there is now evidence in favour of anti-PD-1 monotherapy and anti-PD-1/CTLA-4 combination therapy as a first line treatment for mCRC; this is reflected in the most recent NCCN guidelines [7]. The introduction of immunotherapy has had a substantial impact on OS and DFS in patients with mCRC [123].

#### 3.3.4. CDX2

CDX2 is a homeobox gene that encodes a transcription protein factor that is a major regulator of intestinal development and differentiation [124]. It has also been hypothesized that CDX2 has a tumour suppressor role in the adult colon [125,126]. Currently, CDX2 is used as an immunohistochemical marker of intestinal epithelium, especially in classifying cancers of an unknown origin [127]. More recently, CDX2 has been identified as an emerging prognostic biomarker in CRC where CDX2 loss has been proven to be an independent risk factor for reduced OS and DFS [128,129,130]. CDX2 is absent in around 10% of CRC cancers [130], and CDX2-negative tumours are often associated with several adverse prognostic features, such as advanced stage, vascular invasion, poor differentiation, right-sided location, CIMP, and BRAF mutation [128,131]. Loss of CDX2 expression in CRC is associated with lower OS and DFS, independent of ethnicity, MSI status, or stage [128,129,130].

A recent systematic review and meta-analysis by Tomasello et al. found that CDX2 expression was associated with 50% lower risk of death compared to poor or no CDX2 expression. This finding is more pronounced in stage II and III CRC, with up to 70% risk reduction in OS. CDX2 expression was also associated with a 52% lower risk of disease recurrence [129].

While several studies have demonstrated an association between loss of CDX2 expression and poor prognosis, the association has been inconsistent between studies. Bruun et al. demonstrated that CDX2 was prognostic only in stage IV and stage III BRAF-mutated CRC patients, and not in stage I, II, and stage III BRAF-wildtype CRC patients [132]. In stage II CRC patients, Slik et al. showed that CDX2 loss was associated with reduced DFS and DSS only in the MSS cohort, not the MSI-H cohort [133]. Some studies failed to find any association between CDX2 and prognosis [134,135].

**Core Tip:** CDX2 is not currently used in prognostication in CRC cancer. Current NCCN, ESMO, NICE, and Australian guidelines do not identify CDX2 as a major factor in prognostication [1,7,9,46,47]. However, recent studies, including level 1 evidence have reported a significant association between CDX2 loss and worse prognosis. CDX2 is a useful immunohistochemical marker of intestinal epithelium and the presence of CDX2 in a tumour of an unknown origin increases the likelihood of a gastrointestinal origin.

### 3.4. Histological Features

#### 3.4.1. Tumour Size

Tumour size in CRC refers to the maximum diameter of the tumour specimen [136]. While it is well established and incorporated into the T staging in other solid tumours, such as those of the breast, lung, and thyroid [6], its prognostication ability in CRC remains controversial [137,138,139]. The current AJCC-UICC T staging in CRC is determined by depth of tumour invasion through the layers of bowel wall rather than tumour size [6].

There have been studies that have reported an association between increased tumour size and poor prognosis [137,139,140,141,142,143,144,145]. A larger tumour size has been associated with other poor prognostic features, such as higher grade, T stage, nodal metastasis, and tumour necrosis [143,146]. In a large population-based study on patients with colon cancer (*n* = 300,386), Saha et al. found patients with a tumour size >6 cm had a 46% increased risk of overall mortality compared to a tumour size of <2 cm after adjusting for grade, nodal status, sex, and age [143]. Similarly, in another large population-based study of colon cancer patients (*n* = 128,369), Feng et al. reported that a larger tumour size increased the hazard ratio of death, reducing overall survival (OS) (HR: 1.026; 95% CI: 1.022–1.030; *p* < 0.05) and cancer-specific survival (CSS) (HR:1.037; 95% CI; 1.032–1.463; *p* < 0.05). Possible reasons for difference in survival based on tumour size may have difficulties in achieving complete resection margins in larger tumours or due to the vertical invasion mechanics of the tumours [139].

On the other hand, several studies have reported that tumour size does not maintain independent prognosticative ability [138,147,148,149,150,151,152,153]. Adverse features are not limited to larger tumours, and smaller tumours with T4b infiltration and/or lymph node metastases may be associated with worse prognosis regardless of tumour size [154,155,156,157,158].

The prognosticative ability of tumour size also seems to vary according to location. Some studies have found a direct relationship with tumour size and poorer prognosis in rectal cancer [140,142,159] while others have not [137,138]. One study reported that tumour size was associated with worse OS and CSS in all CRC except tumours in the rectosigmoid junction [140]. Kornprat et al. reports differing cutoffs for optimal prognostication of tumour size according to anatomical location, with decreasing cutoffs from right to left [137].

Variable size cutoffs used by different studies contribute to the ongoing heterogeneity of evidence surrounding prognostication of tumour size in CRC [160]. There is currently no consensus cutoff value for tumour size in international guidelines. Several studies have suggested a tumour size cutoff of <4 cm/≥4 cm to be of prognostic value. Variable reporting and analysis in studies assessing tumour size, such as analysis of diameter as a continuous variable [139,141], the use of receiver operating characteristic (ROC) statistics [145], and X-tile programming [154] have resulted in a range of results.

**Core Tip:** While tumour size is commonly recorded by pathologists, it is not incorporated into the current AJCC-UICC TNM staging system for CRC [6]. It does not currently determine management in any international guidelines [1,7,46,47,122]. Tumour size ≥ 4 cm may be associated with worse prognosis if associated with other adverse pathological features or incomplete surgical margins, but there is not conclusive evidence that tumour size alone is an independent prognostic feature.

#### 3.4.2. Tumour Budding

Tumour budding is a histological finding that represents the dissociation of malignant cells from the invasive front of the tumour [161]. A tumour bud is defined as a cluster of one to four tumour cells at the invasive front of CRC, and is reported using a three tier system based on the normalised number of tumour buds [162]. Recent studies have reported that tumour budding may be an independent prognostic biomarker in colorectal cancer patients. Its potential to identify high risk stage II CRC patients who would benefit from adjuvant chemotherapy has also been reported [161,162,163,164]. The evidence and prognostic utility in tumour budding are highest in stage I and II CRC patients, where it may allow the identification of CRC patients at risk of nodal metastasis [165,166,167]. Due to its novel nature and the relatively recent establishment of an international consensus definition, tumour budding is not widely used in clinical practice [162]. However, the utility of tumour budding has been recognised in some recent clinical guidelines [46,47]. Tumour budding has also been associated with other aggressive pathological features including nodal metastasis, competent mismatch repair (MSS), venous invasion, and poor tumour differentiation [161,166,168].

Several studies and systematic reviews have found tumour budding to be independently associated with disease recurrence, cancer-related death, and reduced OS [164,168,169,170,171]. A comprehensive review by Lugli et al. in 2020 demonstrated worse prognosis in the setting of higher stage tumour budding in multivariate analysis (5-year DSS 89–98% vs. 52–80% in low-grade vs. high grade BD1 vs. BD2–3) [163]. Koelzer et al. demonstrated that tumour budding is associated with poorer OS and DFS following curative resection for stage II CRC [161]. Similar findings have been demonstrated in patients with rectal cancer and high grade tumour budding [172].

While the value of tumour budding in CRC prognostication seems to be most apparent in early CRC [168], the worse prognostication of tumour budding applies to all stages of CRC. In stage III CRC, Yamadera et al. also showed a significant association between high grade tumour budding and chemoresistance [173]. In the metastatic setting, Nagata et al. showed a 5-year survival rate of 18.4% for BD3 compared with 40.5% for BD 1 or 2 (HR 1.51, *p* < 0.009) in patients with metastatic CRC [171].

However, several studies have shown that the prognostic significance of tumour budding was not significant on multivariate analysis [162,174]. Sy et al. questioned the utility of tumour budding in the prognostication in CRC patients with nodal metastases, suggesting that once the tumour has spread to lymph nodes, the degree of tumour budding is less important for the subsequent biological behaviour of the tumour and therefore provides little additional prognostic information in stage III and IV disease [174].

Until recently, the application of tumour budding in clinical practice was limited by a lack of standardised assessment and reporting methodology. In 2016, the International Tumor Budding Consensus Conference (ITBCC) reached a consensus on an international, evidence-based standardised scoring system for tumour budding in CRC [162]. According to the criteria, tumour budding is stratified into three categories: BD1 (low, 0–4 buds), BD2 (intermediate, 5–9 buds), and BD3 (high, ≥10 buds). Subsequently, the literature has validated the association between intermediate/high grade tumour budding with adverse clinicopathological features [162] and worse RFS and OS [163,165,167].

Strong correlations between KRAS/BRAF-mt and tumour budding have been reported [175]. Furthermore, patients with mCRC with tumour budding and/or KRAS-mt respond poorly to anti-EGFR therapy [176].

**Core Tip:** Tumour budding was added as a potential tumour-related prognostic factor in the Union for International Cancer Control (UICC)’s 8th edition of TNM Classification of Malignant Tumours in 2017, a non-core component of pathologic staging of CRC [6]. The prognostic relevance of tumour budding is reflected in the latest publications by the Union for International Cancer Control (UICC), as well as its inclusion in the guidelines for CRC screening, diagnosis, and treatment in Europe and Japan [1,6,177]. ESMO guidelines have listed tumour budding as a high risk feature (along with lymphatic or venous invasion and grade 3 differentiation) [1]. However, tumour budding is not currently included as a high risk adverse feature in several guidelines, including NICE guidelines [8,9].

#### 3.4.3. Tumour Location

Right-sided and left-sided CRC have different clinical and biological profiles [178]. The right colon is derived from the embryonic mid-gut, while the left-sided colon and rectum are derived from the hind gut [179]. There may be differences in the carcinogenic pathway for right- and left-sided CRC [11]. Patients with right-sided CRC are more likely to be female, have a higher median age of diagnosis, are more likely to have high grade histology, and higher tumour stage at initial presentation compared to patients with left-sided CRC [100,178,180,181]. Patterns of metastases also appear to differ according to location: Right-sided CRC tend to metastasize to peritoneum and a greater proportion of left-sided CRC has a tendency to metastasize to liver and lung [179]. The evidence for right-sided colorectal cancer as a poor prognostic factor is strongest in metastatic CRC (mCRC), although it may also be useful in prognostication in the non-metastatic setting [100,158,179,181,182,183].

In mCRC, studies have shown that patients with right-sided tumours have worse than those with left-sided tumours [183,184,185,186]. The primary tumour location is predictive of prognosis and outcome in mCRC [184,187]. A multivariate analysis of a prospective pharmacogenetic study (PROVETTA) and two randomised phase III studies (AVF2107g & NO16966) by Loupakis et al. in 2014 examined over 2000 patients with previously untreated mCRC [183]. Superior OS and PFS were observed in patients with left-sided mCRC compared with right-sided mCRC across all three studies. This was independent of the BRAF status. A systematic review by Stintzing et al. in 2017 examined 10 studies on mCRC, concluding that prognostication of tumour-sidedness was independent of mutational status (KRAS and BRAF) [179]. The difference in OS according to tumour sidedeness may be explained by the difference in recurrence patterns [188]. Peritoneal metastasis may be more difficult to control than liver or lung metastasis [189], and worse prognostication of right-sided CRC may be explained by its tendency to metastasize to the peritoneum [188,189].

In the non-metastatic setting, the literature also suggested worse prognosis in right-sided CRC. A systematic review and meta-analysis of 66 studies in 2016 by Petrelli et al. compared the OS of right-sided CRC to left-sided CRC in over 1.4 million patients in all stages of CRC. Left-sided CRC had improved OS compared to right-sided CRC with a pooled HR of 0.82 (*p* < 0.001), independent of cancer stage, study type, and race [178]. A population study of CRC (*n* = 311,239) by Zheng et al. also demonstrated poorer overall survival in right-sided CRC (right-sided OS 56.1%, left-sided OS 60.2%, HR 1.224, *p* < 0.001), regardless of stage [181]. In stage III CRC, several studies have shown right-sided CRC to be associated with significantly shorter CSS after recurrence compared to left-sided CRC [188,190].

BRAF/KRAS mutations are more common in right-sided CRC [178,191,192]. Studies have found poorer OS in mutant KRAS and BRAF tumours in patients with locally advanced [193] and metastatic [95] disease. Therefore, the worse prognosis in right-sided mCRC and stage III CRC may be partially explained by the higher incidence of KRAS and BRAF mutations [179,191].

On the other hand, right-sided CRC may have a better prognosis in early stage disease [158,192,194,195]. This may be due to a higher incidence of immunogenic MSI-H CRC on the right side. A large population-based study in 2017 by Wang et al. examining 33,789 stage II CRC patients showed that overall cancer specific survival was higher in right-sided CRC compared to left-sided CRC and rectal cancer in both univariate (86.5% vs. 83.8% and 78.7% respectively, *p* < 0.001) and multivariate analysis (HR 0.642 vs. 0.760, *p* < 0.0001) [195]. An analysis of 899 stage II and III CRC patients by Fukata et al. showed improved RFS in patients with stage II right-sided CRC [194]. In a population study of 53,801 CRC patients, Weiss et al. found that right-sided stage II CRC had lower mortality than left-sided stage II CRC (HR 0.92, *p* = 0.001) but higher mortality in stage III disease (HR 1.12, *p* < 0.001) [196]. Similar to Weiss et al., A 2019 Japanese population-based study demonstrated that prognosis in right-sided CRC is worse than left-sided CRC for stage III and IV colon cancer, however is superior in stage I disease [180].

There is not a clear definition of right- and left-sided colorectal cancer. Some studies define cancers located from the rectum to the splenic flexure colon as left-sided cancers, whereas those from the splenic flexure colon to the cecum as right-sided cancers [195,197]. By contrast, other studies have defined right-sided cancers as involving only cecum and ascending colon [188]. Some did not include rectal cancers as part of left-sided CRC [180,190,196,198]. Shida et al. examined 9194 stage III CRC patients and separated them into three groups: Rectal, left-sided, and right-sided CRC (as opposed to left- versus right-sided). They found that RFS rates were similar between right-CRC and left-sided CRC, and that rectal cancer had worse RFS (70%, 69.3%, and 58.4%, respectively, *p* < 0.001). However, OS after recurrence was worst in right-sided CRC followed by rectum, then left-sided colon [198].

There are differences in biomarkers including BRAF, KRAS, and MSI in right- and left-sided CRC. MSI-high tumours are mainly seen in the right colon and carry a favourable prognosis and stage profile [180,188]. They are also less likely to disseminate (stage III and IV disease) [188,199,200]. Thus, improved prognosis in early stage right-sided CRC could be explained by a higher proportion of MSI-H patients, worse prognosis in late stage right-sided CRC by increased BRAF-mt and KRAS-mt CRC.

**Core Tip:** In mCRC, tumour location has a strong prognostic value. Australian and NCCN guidelines state that while anti-EGFR therapy is recommended in left-sided CRC, the recommendation for anti-EGFR therapy in right-sided mCRC should be individualised on a case-by-case basis, given its lack of proven benefit [7,9]. Primary tumour location is not a major consideration in international guidelines in non-metastatic CRC [1,8,10,122].

#### 3.4.4. TILS

TILs is a histological finding that represents a patient’s immunogenicity which is believed to be protective against tumour progression [136,201]. TILs mediate recruitment, maturation, and activation of immune cells that suppress tumour growth [202]. Subtypes of lymphocytes reported to influence CRC outcomes include subtypes of T lymphocytes (CD3, CD4, CD8, CD45R0, and FoxP3 cells), natural killer (NK) cells, and macrophages [203]. Studies have shown that TILs are a positive prognostic factor in CRC, independent of traditional histologic tumour grades. High density TILs are associated with prolonged OS, CSS, and DFS [203,204]. Higher density TILs are also associated with favourable tumour characteristics, such as lower rates of vascular invasion, lymphatic invasion, perineural invasion, lymph node, and distant metastases [201]. TILs have been shown to have a positive effect on prognosis/survival [205]. CD3, CD8, and FoxP3 subtypes of TILs have been shown to have greatest benefit in terms of prognostication [203,206].

A systematic review and meta-analysis of 43 studies by Idos et al. [203] in 2020 revealed that higher generalised TIL density was associated with an improved OS (HR = 0.65; 95% CI, 0.58–0.77), CSS (HR = 0.58; 95% CI, 0.46–0.73), and DFS (HR = 0.72; 95% CI, 0.60–0.88). Specific subsets of lymphocytes were also analysed (CD3, CD4, CD8, CD45R0, and FoxP3 cells) within different tumour locations (tumour center, invasive margin, and stroma). Of all the lymphocyte subsets, the authors demonstrated generalised TIL count and CD3 subsets to have the strongest association with survival benefit. Other T-cell subsets trended towards a favourable prognosis, however there was variability across studies and by tumour location. CD3 and CD8 density has been reported as an independent prognostic factor with Eriksen et al. demonstrating that higher CD3 and CD8 counts were associated with improved RFS (HR = 1.39, *p* = 0.026 and HR = 1.39, *p* = 0.32 respectively) and OS (HR = 1.53, *p* = 0.004 and HR = 1.59, *p* = 0.003 respectively) in stage II CRC patients (*n* = 573) [207].

While TILs have been associated with favourable survival outcomes, the prognostic effect of each TIL subtype is variable [205]. A systemic review of macrophages and FoxP3 cells showed that while they were associated with improved survival on the whole, there is more inconsistency in the literature, with some studies citing a positive influence on prognosis and others citing a negative influence [203,205]. Additionally, there is currently no standardised method of evaluating TILs across studies. A standardised method of TIL evaluation is required to improve consistency and reproducibility of TIL measurements for future diagnostic studies [201,203].

**Core Tip:** The value of TILs in prognostication of CRC is based on its immunogenicity. Novel immunological scoring systems for CRC, such as the Immunoscore looks at TILs at the core and invasive margin of tumour. The Immunoscore is a summation of scores of the two regions (the core of the tumour and the invasive margin) using immunohistochemistry to identify CD3+ and CD8+ T lymphocytes [208]. Initially conceptualised by Galon et al. [209], recent studies have validated the Immunoscore in a large prospective cohort of >2500 CRC patients and demonstrated its prognosticative ability to be equivalent to or more accurate than the conventional TNM staging system [206,208,210]. Currently, TILs assessment is not commonly used in the clinical setting for prognostication [1,7,8,9,47,122]. However, recent studies have shown promise, and TILs may be a useful pathological feature to guide adjuvant treatment including immunotherapy [203,206]. Recent updates to international guidelines have incorporated immunoclassification alongside established TNM staging in predicting prognosis and recurrence in CRC. The latest (5th) edition of WHO Digestive System Tumours introduced the immune response as an essential prognostic criteria for colorectal cancer [211], and ESMO guidelines discuss the role of Immunoscore in determining the risk of recurrence as well using Immunoscore to tailor adjuvant decision-making in challenging cases [1].

#### 3.4.5. Lymph Node Yield

Lymph node yield (LNY), defined as the number of lymph nodes retrieved following specimen dissection, [136] is a strong prognostic factor, particularly in non-metastatic CRC [38,39,41,212,213,214]. Inadequate LNY may be a factor in decision-making for adjuvant chemotherapy [1,7]. Several studies have demonstrated that a higher LNY, regardless of status (positive or negative) is associated with improved OS, DFS, and reduced risk of recurrence [39,41,215,216]. LNY has been established as an important prognostic factor in stage II and III colorectal cancer [39,41,212,214] and while its utility in stage I CRC is less clear [214], emerging evidence suggests that there is a prognostic association in stage I CRC [38,39,213].

A systematic review by Chang et al. in 2007 concluded that increased LNY was associated with improved survival in stage II and III CRC [39]. A retrospective study by Foo et al. examining 659 stage I and II CRC patients showed that a lymph node yield of ≥20 was associated with improved DFS (HR 0.358, *p* = 0.007) and 5-year OS (78.9% vs. 68.2%, LNY ≥ 20 vs. LNY < 20 respectively, *p* = 0.036) [213]. Foo et al. also showed that improved survival with higher LNY was most pronounced in the stage II cohort. Backes et al. showed that a LNY of ≥10 was associated with a decreased risk of recurrence (HR 0.2, *p* = 0.009) in T1 CRC. There is also emerging evidence that an increasing LNY is associated with improved survival in synchronous CRC [217].

In stage III rectal cancer, neoadjuvant therapy is the current standard of care, and a reduction in lymph node yield secondary to radiotherapy is well recognised [218]. Evidence suggests that a reduced yield in this setting may not necessarily confer a poorer prognosis [219], although several studies have demonstrated worse survival [220,221].

There is a lack of consensus on what defines an adequate LNY [212,214]. LNY has multiple influences, including surgical, patient, and laboratory factors [136]. The AJCC/UICC require at least 12 nodes for adequate staging, based on a study demonstrating that this was adequate to determine node positivity in 94% of specimens [222]. It is believed that there may be diminishing returns for staging accuracy beyond 12–17 lymph nodes [40,223]. It remains uncertain as to how increased LNY improves outcomes in CRC. One theory may be that a higher number of retrieved lymph nodes may lead to an improved detection of node positive patients (known as the stage migration effect) [214]. However, multiple studies have shown that despite an increase in lymph node yield over time, the proportion of stage III CRC remains largely unchanged [223,224,225]. Another possibility is that increased LNY improves the clearance of occult micrometastatic disease that would otherwise have been undetected in routine pathological examination [226]. Another theory postulates that LNY correlates with the patient’s immune response to cancer, with a greater abundance of lymph nodes a sign of an effective immune response leading to improved prognosis and survival [227].

**Core Tip:** LNY is currently used in multiple international guidelines for prognostication and CRC management. The AJCC/UICC staging manual and other international guidelines recommends that a minimum of 12 lymph nodes should be identified in colorectal cancer specimens for accurate determination of lymph node involvement for staging purposes [1,6,8,9,47,136]. Reduction in LNY secondary to radiotherapy for rectal cancer is well recognised. In this setting, decreased LNY may not be associated with worse prognosis.

#### 3.4.6. Lymph Node Ratio

Lymph node ratio (LNR) is defined as the ratio of the number of positive lymph nodes to the number of lymph nodes examined histologically [228]. A higher LNR is associated with a more advanced T stage, lymph node metastases, and distant metastases [229]. LNR may provide an indication of tumour behaviour, extent of surgical resection, and host immune response [230]. LNR is only pertinent to stage III and IV CRC as positive lymph node is associated with at least stage III disease [6]. The role of LNR in CRC was initially studied by Berger et al. in 2005 [231]. LNR has been reported as a negative independent prognostic indicator in stage III and IV CRC and is associated with reduced OS and DFS [33,44,229,230,232,233,234,235]. A systematic review and meta-analysis by Ceelen et al. in 2010 reported a pooled HR of 2.36 for OS and 3.71 for DFS in stage III CRC patients with a high LNR [230]. A subsequent systematic review and meta-analysis by Pyo et al. of 14 studies confirmed this finding. LNR has a stronger negative association in rectal cancer than colon cancer [232]. Several studies demonstrated that LNR is superior to the number of positive nodes (current N staging) in both stage III and IV CRC [230,232,234]. The prognostic significance of LNR may be greater when there is a lymph node yield <12 [44,236]. Evidence for LNR as an independent prognostic factor is strongest in stage III CRC, however evidence for stage IV CRC is also robust [33,229,233].

The cutoff for high LNR varies from 0.125 to 0.3 in different studies and is defined differently across international guidelines [230,232]. In all 16 studies examined by Ceelen et al., LNR was analysed as a categorical variable. Some studies constructed LNR quartiles based on the distribution frequency, others classified LNR categories based on maximal separation of survival curves [230]. One study adapted classification and regression trees to define optimal LNR cutoff points [235]. There is no consensus on the minimum required number of harvested lymph nodes for proper evaluation of LNR [44,214].

**Core Tip:** AJCC-UICC TNM staging defines N stage by the number of affected regional lymph nodes, not LNR [6]. While several studies have shown that LNR may be an independent prognostic factor, LNR does not currently play a role in N staging, nor is it used in any international CRC guidelines [1,7,8,9,10,46,47]. There is no consensus on cutoff values for LNR for prognostic significance and the minimum required number of harvested lymph nodes for proper evaluation of LNR.

#### 3.4.7. Apical Lymph Nodes

Apical lymph nodes (ALN) refers to lymph nodes within the origin of the major vessel that supplies the tumour [228]. ALN involvement occurs in 4–19% of CRC patients and is associated with higher rates of nodal invasion, deeper tumour infiltration (T3/4), and para-aortic nodal recurrences [43,237,238,239]. The role of ALN metastasis in prognostication is controversial [237,238,239,240,241]. Some studies have shown it to be an independent predictor of poorer OS and DFS [42,43,237,240]. Tsai et al. found ALN involvement to be more predictive of distant metastasis post-operatively compared to regional lymph node metastasis [43]. Huh et al. [242] and Kim et al. [237] found that stage III CRC patients with ALN involvement had similar survival rates compared with stage IV disease with R0 resection, and postulated that ALN metastasis should be considered systemic rather than regional. Other studies failed to prove an association between ALN metastasis and long-term survival and suggested that it be considered a regional metastasis [238,239,241,243]. Wang et al. utilised propensity score matching to reduce baseline bias between patient groups (ALN positive versus ALN negative) and found that the ALN status was not a significant risk factor for survival in both right- and left-sided CRC [238,239].

There are limitations in the current literature on the prognostic ability of ALN metastasis. There is currently no consensus on its definition [244], for example, in left-sided CRC, some studies define ALN as nodes within 1 cm of the inferior mesenteric artery (IMA) origin, [42,43,238,239] while others define the ALN as nodes from the origin of the IMA to the takeoff of the left colic artery (LCA), which would usually exceed 1 cm [240,241,243]. Additionally, patient baseline characteristics varied between studies and the absolute numbers of ALN-positive patients are often low [245].

**Core Tip:** ALN status is utilised in the Japanese and Australian classification system for CRC [9,10]. The Japanese Society for Cancer of the Colon and Rectum (JSCCR) guidelines for CRC defines lymph node staging by the location of the positive nodes rather than the number of metastatic lymph nodes, in which apical lymph nodes are classified as N3, or LND 3 to avoid confusion with the TNM staging system [10,245]. The Australian classification system, known as the Australian clinicopathological staging (ACPS) system, defines the presence of an involved ALN as the ACPS substage C2 [83]. Current Australasian pathology reporting guidelines recommend the reporting of ALN status [228]. ALN positivity (classified as N3) was utilised in the 3rd and 4th AJCC TNM staging system, however it was subsequently removed due to the complexity of dividing lymph node zones for pathologic evaluation and mixed evidence regarding prognostic benefit [43]. The 8th edition defines ALNs as regional lymph nodes and nodal staging is based on the number of metastatic lymph nodes [6].

#### 3.4.8. Perineural Invasion

Perineural invasion (PNI) refers to the neoplastic invasion of nerves by tumour cells as a method of tumour spread [246]. Tumour cells can grow within, around, and through any of the three nerve layers [246]. The reported incidence of perineural invasion in CRC ranges from 9% to 30%, and occurs more frequently in a higher stage of CRC. Studies have reported PNI in approximately 10% in stage I-II disease, up to 30% in stage III disease, and up to 40% in stage IV disease [246,247,248]. There is evidence that PNI is an independent marker of poor outcome and decreased survival [246,247,249,250]. A systematic review and meta-analysis by Knijn el at. Examined 58 studies with 22,900 CRC patients of all stages. PNI was associated with reduced 5-year DFS (HR 2.35, 95% CI 1.97–308), CSS (HR 1.91, 95% CI 1.56–2.42), and OS (HR 1.85, 95% CI 1.63–2.12). In addition, the prognostic value of PNI was found to be similar to other established prognostic factors, such as depth of invasion, tumour grade, lymph node metastasis, and extramural invasion [247]. These findings are further highlighted by a large Surveillance, Epidemiology, and End Results (SEER)-based population study of 41,000 CRC patients. PNI was associated with a reduced 3-year OS and CSS (HR 1.24 and HR 1.28 respectively, *p* < 0.001), independent of T stage, N stage, tumour grade, and location [251].

There are a lack of uniform reporting standards and guidelines for PNI [136]. PNI tends to be underreported, with detection rates ranging from 9% to 42% [247,249]. Various definitions of PNI are used across multiple studies [246]. Tumour cells surrounding >33% of the nerve circumference is one of the more commonly-used definitions in the literature [246].

**Core Tip:** Documentation of PNI status is part of standard CRC pathology reporting [136,228]. The AJCC-UICC 8th edition of TNM staging identifies PNI as an additional tumour-related prognostic factor [6]. Level 1 evidence, including meta-analysis and large database studies have demonstrated that PNI is associated with worse prognosis but there needs to be better standardisation of PNI reporting.

#### 3.4.9. Circumferential Resection Margin

Circumferential resection margin (CRM) is also known as the radial margin, mesenteric margin, or non-peritonealised margin. It is the distance (in millimetres) between the deepest point of tumour invasion and the surgically dissected non-peritonealised surface of the specimen [136,228]. Low rectal tumours below the peritoneal reflection are completely surrounded by this circumferential, non-peritonealised margin while upper rectal tumours have a non-peritonealised margin posterolaterally and a peritonealised surface anteriorly [228]. For colonic tumours, the mesenteric attachment of the colon and cut edge of the retroperitoneal segments form the circumferential margin [136].

Criterion used to define a positive CRM remains controversial [252,253,254]. The most commonly-used definition of CRM positivity is tumour ≤1 mm from the tumour-free margin [252]. CRM positivity occurs in 7.3% to 25% of rectal cancers and 5.3% to 20.5% of colon cancers. It is associated with advanced stage, aggressive tumour grade, infiltrating tumour border, and lymphovascular and perineural invasion [253,255]. In rectal cancer, CRM positivity is a strong predictor of recurrence and reduced survival, independent of TNM staging [252,256,257,258,259]. Positive CRM is associated with increased risk of local recurrence (HR 4.67 95% CI 2.51–4.15), distal metastasis (HR 2.95), and reduced OS (HR 3.21) and DFS (HR 3.63) [252]. The prognostic significance of CRM is stronger in patients undergoing neo-adjuvant radiotherapy prior to surgery compared with surgery alone, likely because tumours with limited response to radiotherapy are biologically unfavourable [256].

The significance of CRM positivity is less studied in colon cancer, however recent evidence suggests that its poor prognostication in rectal cancer also applies to colon cancer [253,254,255,260]. Amri et al. initially confirmed the prognostic significance with CRM involvement in colon cancer in 2015, identifying it as an independent prognostic factor linked with reduced OS (HR 3.39 *p* < 0.001), reduced DFS (HR 2.03 *p* < 0.001), and higher rates of recurrence (HR 3.32 *p* < 0.001) [253]. These findings are confirmed in a large population study by Tang et al. in 2020, which found that patients with a CRM value of 0–30 mm benefited most from chemotherapy [255]. Evidence on the optimal CRM in both rectal and colon cancer is inconsistent. Multiple thresholds of CRM clearance have been proposed. In rectal cancer, a CRM clearance of greater than five millimetres was proposed by Kelly et al. in 2011, and <0.4 mm proposed by Beaufrere et al. in 2017 [261,262]. In a large 2018 population study, Liu et al. divided CRM groups in rectal cancer patients into 0–1 mm, 1.1–2.0 mm, 2.1–5 mm, 5.1–10 mm, and >10 mm and examined survival outcomes between the subgroups. There was a survival benefit for the CRM 5.1–10-mm group compared to the 1.1–5-mm group, however this was not statistically significant [258]. In colon cancer, Tang et al. found a margin >30 mm was associated with improved outcomes among CRM-negative patients [255].

**Core Tip:** Documentation of CRM involvement is standard in pathological reporting of CRC [136,228]. International guidelines recommend negative margins (>1 mm) for all CRC patients undergoing resection, and the rate of CRM positivity is widely used as a quality indicator in rectal cancer surgery [1,7,9,10]. A positive CRM denotes at least T3 disease with an R1 or R2 resection in the AJCC-UICC staging manual [6]. NCCN guidelines include CRM positivity as criteria for adjuvant chemotherapy in stage II CRC [7], and ESMO guidelines recommend its consideration for risk assessment in these patients [1]. An expanded R classification that considers minimal distance between tumour and a resection margin has been proposed by Wittekind et al., but is currently not adopted by the AJCC-UICC TNM staging manual [6,254].

#### 3.4.10. Tumour Grade

Tumour grade or histological grade refers to the degree of tumour differentiation and is an adverse prognostic factor independent of stage in CRC [263,264,265,266], with reduced DFS, reduced DSS, and increased risk of recurrence [18,108,147,265,266]. Higher tumour grade is also associated with an advanced stage, increased tumour invasion depth, positive nodal status, and lymphovascular invasion [263,267]. Stage I and II CRC patients with higher tumour grade may have worse DSS than stage III disease with a low tumour grade [267].

The main limitations in tumour grading lie in the lack of a single, uniformly used system and significant interobserver variability in its assessment [263,265]. There is variation on whether it should be based on the predominant pattern of differentiation or the area of least differentiation [263,267]. Most definitions of tumour grade are based on the percentage of gland formation; the inclusion of cytologic or other features in the estimation of grade is variable [264]. The college of American pathologists (CAP) use a four-tiered grading system for CRC, based solely on the degree of gland formation [136]. Grade 1 is classified as well differentiated (>95% gland formation), grade 2 moderately differentiated (50–95% gland formation), grade 3 poorly differentiated (<50% gland formation), and grade 4 undifferentiated (no gland or mucin formation) [136]. The World Health Organisation (WHO) classification of digestive system tumours uses a two-tiered system: Low grade (≥50% gland formation) and high grade (poorly differentiated, <50% gland formation) [211]. In 2012, Ueno et al. proposed a novel grading system based on clusters of ≥5 cancer cells lacking a gland-like structures (termed poorly differentiated clusters), [265] with some evidence of improved reproducibility and prognostic power [264,267].

**Core Tip:** Tumour grade is a part of most international guidelines on CRC as well as in clinical practice. Histological grading is included in the histopathological reporting of CRC in routine practice, and is classified as an additional prognostic factor in the AJCC-UICC TNM staging manual [6,228]. Poorly differentiated tumours are more likely to be referred for adjuvant chemotherapy in stage II CRC [4]. Multiple international guidelines (ASCO, NCCN, ASCRS, NCI, ESMO, and NHMRC) identify poorly differentiated tumour grade as a risk factor for recurrence in stage II CRC and suggest consideration of adjuvant chemotherapy when present [1,7,8,9,10,122].

#### 3.4.11. Lymphovascular Invasion

Lymphovascular invasion (LVI) refers to the involvement of small lymphatic or blood (typically venous) vessels by tumour on histological examination [136]. LVI is considered a key step in the development lymph node metastasis [268]. The incidence of LVI in CRC has been reported to vary from 4.1% to 63.8%, likely due to different study populations and diagnostic techniques used [268,269]. LVI has emerged as a well-recognised stage-independent predictor of poor prognosis in CRC [248,268,269,270,271,272,273]. Several systematic reviews and large-scale population studies have shown that LVI-positive CRC patients have up to a 55% decrease in OS and significantly reduced DFS (HR 1.73 CI 1.50–1.99 *p* < 0.01) [248,268,269,272,273]. LVI is associated with other adverse features, such as a higher pathologic tumour stage, lymph node involvement, distant metastasis, poor differentiation, larger tumour size, perineural invasion, tumour budding, and positive KRAS status [248,268]. The poor prognostication of LVI applies to all stages of CRC [248]. Patients with node negative disease, especially stage II, are the most likely group to benefit from identification of LVI [248,269,272].

**Core Tip:** Given its prognostic significance in CRC, LVI is currently classified as an additional prognostic factor in the AJCC-UICC TNM staging manual [6]. Pathological assessment of LVI is recommended by the College of American Pathologists [136]. Currently, there is no accepted standard for LVI in pathological reporting [248]. There is significant interobserver variability in the diagnosis in LVI, and there is some evidence to suggest that different modalities of staining (such as elastic stains and immunohistochemical markers) can influence the accuracy rate [136,274]. As such, true rates of LVI are likely higher than currently reported [274]. While invasion of extramural veins is an independent predictor of poor outcome and increased risk of hepatic metastasis, the significance of intramural venous invasion is less clear [275].

## 4. Conclusions

A complex interaction of stage, pathological features, and biomarkers play a role in guiding prognosis, risk stratification, and guiding neoadjuvant and adjuvant therapies. Traditionally, the TNM stage has been the main classification system guiding prognosis, neoadjuvant, and adjuvant therapy. However, in the recent decade, greater emphasis on molecular biomarkers such as MSI, KRAS, BRAF, and CDX2 and histological features such as tumour budding, TILS, CRM, PNI, LVI, apical lymph node involvement, LNY, and LNR has allowed better characterisation of the tumour, improving the accuracy of prognostication as well as the optimisation of neoadjuvant and adjuvant treatment. With the move towards precision oncology, understanding the pathological as well as the genetic features of each tumour and how it impacts treatment and survival may lead to more specific prognostication and treatment protocols tailored to the unique pathological and genetic characteristics of each tumour rather than prognostication and treatment being based mainly on the TNM staging classification.
